# The complete mitochondrial genome of *Beauveria lii* (Hypocreales: Cordycipitaceae)

**DOI:** 10.1080/23802359.2021.1875917

**Published:** 2021-02-12

**Authors:** Sheng-li Zhang, Shun-chang Pu, Ai-ting Lin, Feng-gang Luan

**Affiliations:** a Institute of Biological Engineering, Bozhou College, Bozhou, China; b Shanghai Biozeron Biotechnology Co., Ltd, Shanghai, China; c College of Landscape and Art, Jiangxi Agricultural University, Nanchang, China

**Keywords:** *Entomogenous fungi*, Hypocreales, *Beauveria*

## Abstract

The mitochondrial genome of *Beauveria lii*, strain RCEF500, was sequenced on the NovaSeq 6000 and the Nanopore Sequencer, and annotated. The genome is 59,014 bp in length, encoding 15 conserved protein-coding genes (PCGs), 2 rRNA genes and 23 tRNA genes. The nucleotide composition of *Beauveria lii* mitochondrial genome was 38.23% of A, 35.81% of T, 11.61% of C, 14.36% of G, 25.97% of G + C content. Phylogenetic analysis confirmed *B. lii* as a member of *Beauveria* (Cordycipitaceae). The mitochondrial genome of *B. lii* will contribute to the understanding of phylogeny and evolution of the genus and family.

*Beauveria* (Hypocreales: Cordycipitaceae) is a cosmopolitan anamorphic genus of entomopathogenic fungi, which has diverse niches including insects, soil and plants (Ownley et al. 2008; Vega et al. 2008; Imoulan et al. 2017). It was first described by Balsamo-Crivelli (Balsamo-Crivelli 1835a, 1835b) under the name *Botrytis bassiana* and changed to *Beauveria bassiana* in 1912 by Vuillemin (1912). Although easily distinguishable as a genus, species identification remains definitely complicated because of the lack of distinctive morphological features. Furthermore, the extensive overlap in conidia shape and dimensions among *Beauveria* species has limited their utility as key taxonomic structures (Rehner et al. 2011; Imoulan et al. 2017). *B. lii* was first found in China and caused an epidemic of *Henosepilachna vigintioctopunctata* (Zhang et al. 2013), so it has a potential of biocontrol for *H. vigintioctopunctata*. Here, we reported the complete mitochondrial DNA sequences of *B. lii* (GenBank accession number:MT818175) for the first time, in order to provide valuable information on the gene contents of the mitochondrial genome for the study of rapid interspecific identification and evolution of *Beauveria.* The strain RCEF5500 was isolated from a larva of *H. vigintioctopunctata* (Coleoptera, Coccinellidae), collected in Xunyi County, Shaanxi, China (108°08′–108°52′ E, 34°57′–35°33′N). The isolated strain RCEF5500 is deposited in the Research Center for Entomogenous Fungi (RCEF), Anhui Agriculture University, Hefei, Anhui, China.

Total genomic DNAs were extracted from mycelia cultured on PDA plates covered with cellophane using Blood & Cell Culture DNA Mini Kit (Qiagen) and sequenced on both the Oxford Nanopore PromethION and Illumina NovaSeq platform. Using minimap2 (Li 2018) to compare the original reads with the mitochondrial database (https://ftp.ncbi.nlm.nih.gov/refseq/release/), the matched reads were extracted. For the extracted mitochondrial data containing the second and third generations sequencing data, the mitochondrial genome was mixed assembled and corrected by unicycler (Wick et al. 2017a, 2017b). The final genome was obtained after correcting by nextpolish (Hu et al. 2020) four times using the second generation quality control data. The assembled mitogenome of *B. lii* was annotated as described previously (Zhang et al. 2017).

The complete mitogenome sequence of *B. lii* is 59,014 bp in length, containing 23091 bp gene total length (39.13% of mitogenome), 35,923 bp intergenic region length (60.87% of mitogenome) and 25.97% of G + C content. There are 15 conserved protein-coding genes (PCGs), 23 tRNAs, 2 rRNA genes and 15 ORFs, which were annotated. Those conserved PCGs includes ribosomal protein S3, ATP6, ATP8, ATP9, NAD1 (1 group IB intron), NAD2 (1 group I intron), NAD3, NAD4 (1 group IC2 and group IB intron), NAD4L, NAD5 (2 group IB introns), NAD6, COB (1 group IB and 1group ID introns), COX1 (8 group IB introns), COX2, COX3. 2 ribosomal RNA subunits (rnl and rns) contain 3 (2 group IC1 and 1 group IA introns) and 0 group I introns respectively. The 23 tRNAs covered 20 standard amino acids and clustered into three clusters containing 20 tRNA genes. Two tRNA genes with different anticodon were found for tRNA-Leu, tRNA-Arg and tRNA-Ser. For the remaining 20 tRNAs, only one gene each was found. All genes are encoded on the heavy (+) strand except 3 ORFs.

Phylogenetic analysis was conducted for the mitogenome and 39 other Hypocreales species using the Maximum Likelihood method and Bayesian method with an integrated tree building software PhyloSuite (Zhang et al. 2020). In PhyloSuite, Iqtree 1.6.8 (Nguyen et al. 2015) was used for Maximum Likelihood method under GTR + F+R4 model, while MrBayes v3.2.6 (Ronquist et al. 2012) was used for Bayesian method under GTR + F + I + G4 model. The phylogenetic tree based on 14 PCGs indicated that the mitogenome of this species was genetically the closest to that of *B. brongniartii*. (Figure 1). It conforms that *B. lii* is a member of Cordycipitaceae and supports *Beauveria* as an independent genus in the family.

**Figure 1.**
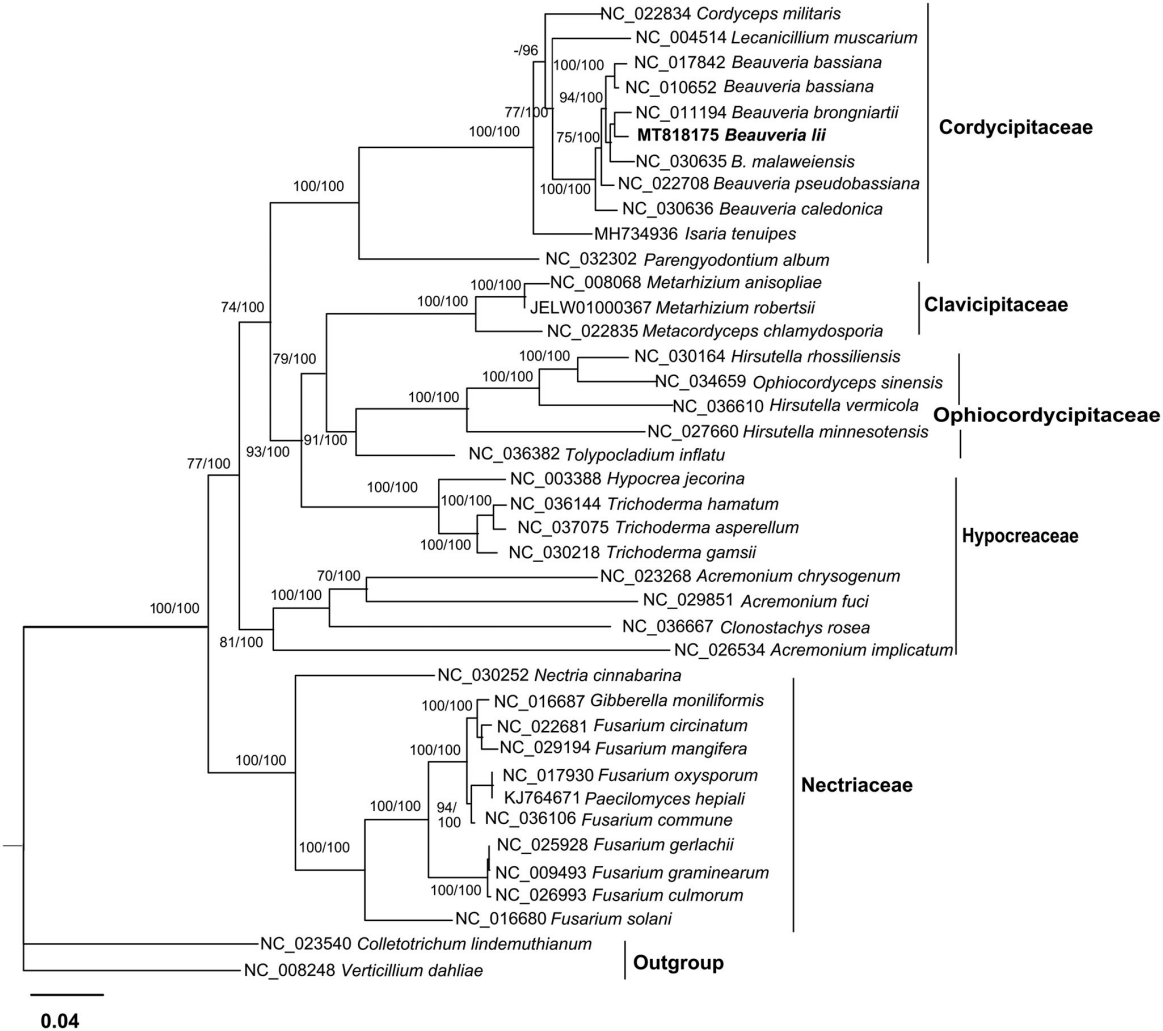
Phylogenetic relationships among 39 Hypocreales fungi inferred based on the concatenated sequences of 14 mitochondrial protein-coding genes. The 14 mitochondrial protein-coding genes were: nad1, nad2, nad3, nad4, nad4L, nad5, nad6, cox1, cox2, cox3, cob, atp6, atp8, atp9. The tree was generated using Maximum Likelihood (ML) and Bayesian method. ML bootstrap values (BS) ≥ 70% and Bayesian posterior probabilities (BP) ≥95%are presented at the nodes.

## Data Availability

The data that support the findings of this study are openly available in GenBank of NCBI at https://www.ncbi.nlm.nih.gov, reference number MT818175.
